# Effect of LPSO and SFs on microstructure evolution and mechanical properties of Mg-Gd-Y-Zn-Zr alloy

**DOI:** 10.1038/srep40846

**Published:** 2017-01-30

**Authors:** Chao Xu, Taiki Nakata, Xiaoguang Qiao, Mingyi Zheng, Kun Wu, Shigeharu Kamado

**Affiliations:** 1 School of Materials Science and Engineering, Harbin Institute of Technology, Harbin 150001, PR China; 2 Research Center for Advanced Magnesium Technology, Nagaoka University of Technology, Nagaoka 940-2188, Japan

## Abstract

High performance Mg-8.2Gd-3.8Y-1.0Zn-0.4Zr alloy with high strength and excellent ductility has been successfully developed by hot extrusion. The effect of plate-shaped long period stacking ordered (LPSO) phases and solute-segregated stacking faults (SFs) on the dynamically recrystallization (DRX) behavior was analyzed. The plate-shaped LPSO phases stimulate the DRX by particle stimulated nucleation mechanism, leading to higher DRX ratio and weaker basal texture. While for the alloy with dense fine SFs inside the original grains, discontinuous DRX initially occurs at the original grain boundaries, and the DRX is obviously restricted. Consequently, alloy containing dense SFs exhibits higher strength but lower ductility compared with alloy with plated-shaped LPSO phases.

In recent years, Mg-Zn-rare earth (RE) alloys containing long period stacking ordered (LPSO) phases have been attracting considerable scientific interest as promising wrought alloys due to their superior mechanical performance^1^,^2^,^3^,^4^. The LPSO phase exhibits strong anisotropy since the (0001) <11
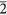
0> basal slip dominates its plastic deformation^5^,^6^. Additionally, the RE and/or Zn enriched atomic layers with stable short range ordered structure in the LPSO phase brings about the higher Young’s modulus (~67 GPa) of the LPSO phase than that of Mg matrix (~40 GPa)^7^,^8^. Therefore, the LPSO phases definitely strengthen the alloy and refine the Mg matrix in the vicinity of the block-shaped LPSO phases due to dynamic recrystallization (DRX) via the particle stimulated nucleation (PSN) mechanism during hot working processes^9^. Yamasaki *et al*.^10^ developed a high strength Mg-Y-Zn alloy by hot extrusion and they reported that both the deformed Mg grains with strong basal texture and block-shaped elongated LPSO phases strengthen the alloy, whereas the dynamically recrystallized (DRXed) Mg grains with very weak texture improves ductility of the alloy. Therefore, control of DRX process play an important role in improvement of properties of Mg alloys.

On the other hand, it is reported that the Mg-Zn-Gd alloy extruded from the billet full of thin lamellar-shaped LPSO phase as well as solute-segregated stacking faults (SFs) shows superior creep resistance than that extruded from the billet containing block-shaped LPSO phases^11^. In addition, the plate-shaped LPSO phases increase the critical resolved shear stress (CRSS) for basal slip, thus the non-basal slip in the α-Mg matrix is activated, which leads to simultaneous improvement of both strength and ductility of Mg-Zn-Y alloy^12^. The block-shaped LPSO phases are reported to promote the DRX process through PSN mechanism^9^. However, the effect of plate-shaped LPSO phases/solute-segregated stacking faults (SFs) inside original α-Mg grains on dynamic recrystallization behavior of the Mg-RE-Zn alloys during hot extrusion has been rarely reported.

The precipitation of plate-shaped LPSO phase and/or SFs can be controlled by heat treatment^13^. In this study, Mg-Gd-Y-Zn-Zr alloys containing plate-shaped LPSO phase or SFs were hot extruded, and the effect of thin plate-shaped LPSO phases and/or solute-segregated SFs on microstructure and mechanical properties of the Mg alloy were investigated.

## Results

### Microstructure of the as-homogenized alloys

**Figure 1:**
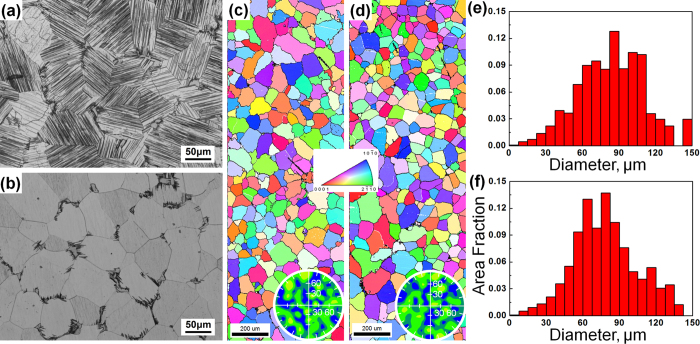
Microstructure observations of the as-homogenized alloys: (**a**,**c**,**e**) furnace cooled alloy and (**b**,**d**,**f**) quenched alloy, (**a**,**b**) optical micrographs, (**c**,**d**) EBSD IPF maps and (0001) pole figures, (**e**,**f**) grain size distribution histograms.

Figure 1a and b shows the optical micrographs of the as-homogenized alloys followed by furnace cooling and quenching. It can be seen that the majority of the eutectic phases are dissolved into the α-Mg matrix and only a small amount of block-shaped LPSO phases with area fractions of about 3% for both alloys remain at the grain boundaries, especially at the triple junctions. The plate-shaped 14 H LPSO phases precipitate and grow across the whole grains during slow cooling process in the furnace cooled alloy^14^, as given in Fig. 1a. While supersaturated α-Mg solution without visible precipitation is obtained in the quenched alloy. In comparison with the quenched alloy, the slow cooling brings no obvious grain growth due to the effective pinning of the grain boundaries by block-shaped phases LPSO and the plate-shaped 14 H LPSO phases inside the grains also restrict the grain growth.

Figure 1 shows the EBSD analysis of the as-homogenized alloys. The average grain sizes are estimated to be about 83 μm and 78 μm, respectively, following a normal distribution. The grains orient randomly as shown in Fig. 1c and d, and accordingly, random texture is obtained for both as-homogenized alloys. Therefore, the initial grain sizes and texture are supposed to be identical for the furnace cooled and quenched alloys so as to benefit the comparison of the effect of plate-shaped LPSO phases and SFs on the microstructure evolution and mechanical properties of the as-extruded alloys.

### Microstructure of the as-extruded samples

**Figure 2:**
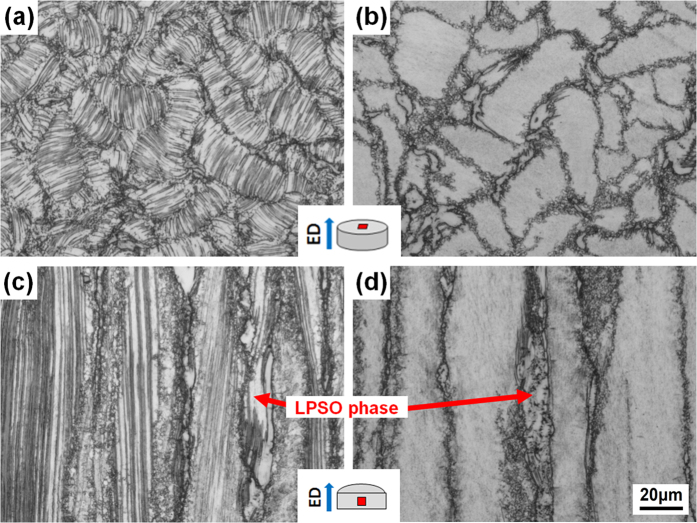
Optical micrographs of the as-extruded alloys observed (**a**,**b**) along extrusion directions and (**c**,**d**) perpendicular to the extrusion directions, respectively. (**a**,**c**) F + E sample, (**b**,**d**) Q + E sample.

Figure 2 shows the optical micrographs of the as-extruded alloys taken from the transverse and longitudinal sections. The undissolved block-shaped LPSO phases are deformed and elongated along the ED. Moreover, the grains are significantly refined and bimodal microstructure consisting of coarse deformed grains as well as fine dynamic recrystallized (DRXed) grains is obtained for both as-extruded alloys. The DRX ratio is obviously higher in the F + E sample than in the Q + E sample (Fig. 2c and d) and the average DRXed grain sizes are estimated to be 1.4 μm for F + E and 1.1 μm for Q + E. It is observed that the fine DRXed grains are mainly formed at the initial grain boundaries and adjacent to the block-shaped LPSO phases. In the F + E sample, a large amount of plate-shaped phases orientated parallel to the ED are observed throughout the whole coarse deformed grains, as given in Fig. 2a and c. While no obvious contrast is visible in the optical microstructure of the coarse deformed grains of the Q + E sample (Fig. 2b and d).

**Figure 3:**
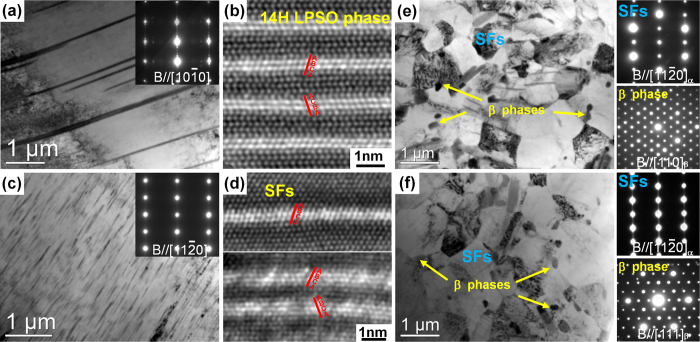
TEM observations of the extruded alloys: (**a**,**c**) and (**e**,**f**) the bright-field images and corresponding SAED patterns taken from the unDRXed and DRXed regions, respectively, **(b,d**) the HAADF-STEM images of the precipitates in (**a**) and (**c**); (**a**,**b**,**e**) F + E sample, (**c**,**d**,**f**) Q + E sample.

As shown in Fig. 3a, the plate-shaped phases observed in the deformed grains of the F + E sample are 14 H LPSO phases with thicknesses in the range from ~30 nm to ~200 nm. This indicates that the plate-shaped 14 H LPSO phases formed during slow cooling process are remained during hot extrusion and reoriented with their basal planes parallel to ED. Figure 3b shows the atomic resolution HAADF-STEM image of the LPSO phase viewed along <11
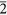
0>_α-Mg_ in F + E sample. The unit cell ABCA of 14 H LPSO phases with Gd/Y and/or Zn atoms segregated in B and C layers contributes to the high elastic modulus of the LPSO phase^7^. Moreover, the two blocks arrange in the opposite shear directions, thus no net shear strain is generated due to the self-accommodation^15^, which leads to superior thermal stability of the 14 H LPSO phase during the hot extrusion process.

As shown in Fig. 3c, the dense thin and short lamellae are observed in the deformed grain of Q + E sample, the streaks along the c-axis in the SAED pattern suggest that they are SFs. Atomic resolution HAADF-STEM images shown in Fig. 3d indicate that the SFs are intrinsic type with stacking sequence of ABAB|CACA or ACAC|BABA. Since the faulted regions are energetically favorable for solute segregation^16^, Gd/Y and/or Zn atoms are enriched in the successive B and C layers. In addition, these SFs are also observed to form in pair and in clusters and the stacking sequence of Gd/Y and/or Zn segregated layers shear in opposite directions for self-accommodation of shear strain, which resembling to that in the 14 H LPSO phase in Fig. 3b. Since no SF is observed inside the grains of the as-homogenized alloy (result is not shown here), these dense SFs should form during heating at 400 °C prior to the extrusion as well as the extrusion process. These SFs will transform into 14 H LPSO phase when increasing heating temperature or heating time^13^,^14^.

BF images of the DRXed regions of the as-extruded alloys (Fig. 3e and f) show that fine equilibrium β-Mg_5_RE particles with average diameters of ~200 nm dynamically precipitate at the DRXed grain boundaries in both alloys during hot extrusion process. The fine β precipitates at the grain boundaries can exert Zener pinning effect to restrict the grain growth. The SAED patterns shown in Fig. 3e and f indicate that fine lamellae inside the DRXed grains are basal SFs formed during the extrusion process. The simultaneous addition of Zn and Gd/Y to Mg alloys can reduce the stacking fault energy (SFE) in Mg alloys, leading to the formation of many faults on the basal planes^13^,^17^. The local strain fields induced by the further deformation after DRX may stimulate the formation of SFs and facilitate the diffusion of solute atoms to these SFs inside the DRXed grains of both as-extruded alloys. Some of the plate-shaped LPSO phases formed during slow cooling process after homogenization are fragmented and mainly distribute at the DRXed grain boundaries of the F + E as indicated by red arrows, which also exert pinning effect and influence the DRX of the alloy during hot extrusion.

### DRX behavior during extrusion process

**Figure 4:**
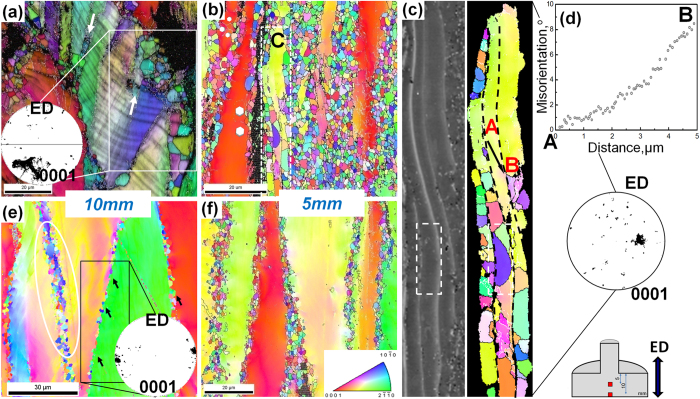
EBSD analysis of the partially extruded alloys: (**a–d**) F + E sample, (**e**,**f**) Q + E sample; (**a**,**e**) IPF maps and corresponding (0001) pole figures calculated from the marked areas taken from the position of 10 mm before the die-entrance, (**b**,**f**) IPF maps taken from the position of 5 mm before the die-entrance, (**c**) magnified IPF map of the area marked C in (**b**) and corresponding SEM micrograph, (**d**) misorientation calculated by point-to-origin along the black arrow in (**c**).

Figure 4 shows the IPF maps at different positions near the die-entrance of the partially extruded F + E and Q + E samples, respectively. During the initiation of the extrusion deformation, DRX preferentially occurs near the block-shaped LPSO phases at initial grain boundaries (corresponding to the shaded area due to the low confidence index^10^) by the PSN mechanism aforementioned, as indicated by white box in Fig. 4a. In addition, the coarse initial grains full of plate-shaped LPSO phases (image quality contrast) in the F + E sample can be observed. The kink bands (KBs) form in the plate-shaped LPSO phases by the avalanche generation of pairs of dislocations^5^, these KBs transfer to the adjacent α-Mg matrix and give rise to the formation of kink grain boundaries (GBs) by synchronized slip of basal dislocation pairs with opposite signs in α-Mg^18^. The dislocations are easy to pile up in the vicinity of the kink GBs and facilitate the DRX at the kink GBs. As a result, a small amount of fine grains with sizes of 1~4 μm form at the kink GBs of α-Mg matrix as indicated by white arrows in Fig. 4a.

**Figure 5:**
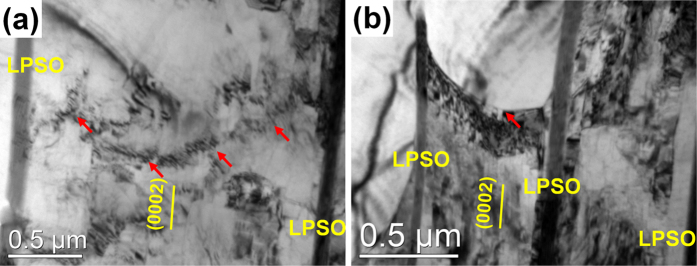
TEM BF images taken from the partially extruded F + E sample (g = 10
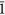
1): (**a**) non-basal <c + a> dislocations pile-ups, (**b**) accumulation of dislocations at the boundaries between plate-shaped LPSO phases.

As the extrusion proceeds, the coarse unDRXed grains elongate along the ED and the volume fraction of the DRXed grains increases obviously (Fig. 4b). The region marked C is magnified and the corresponding SEM micrograph are given in Fig. 4c. It can be seen that plate-shaped LPSO phases in the original grains are retained and orientated to the ED during further deformation. The DRXed grains are clearly observed to form in the α-Mg bands separated by the plate-shaped LPSO phases, thus the DRXed grain size depends on the interspacing of the plate-shaped LPSO phases. The formation of LPSO phase consumes the solute atoms in the α-Mg bands among LPSO phases. So the dynamic precipitation of the β phases is restricted and grain growth occurs for some new DRXed grains as highlighted by white dashed boxes in Fig. 4c. It is noteworthy that color variation can be observed in the unDRXed areas, which indicates the existence of dense dislocations and subgrain boundaries. Figure 5a shows the TEM BF image of the F + E sample at the position of 5 mm before the die-entrance. Non-basal <c + a> dislocations are activated and pile up among the plate-shaped LPSO phases, as marked by red arrows. It is reported that stress concentration is generated at the interface between the plate-shaped LPSO phases and α-Mg matrix due to the elastic modulus mismatch, which can result in a strong portioning of stress to the LPSO phases with high Young’s modulus and of strain to the soft α-Mg matrix^19^. As a result, < c + a> dislocations are supposed to generate at the interface to accommodate the strains along c axis of α-Mg matrix then enhance the DRX process. Figure 4d shows the misorientation gradient approaching the interface between the plate-shaped LPSO phase and α-Mg matrix (AB along the black arrow in Fig. 4c). The misorientation gradually increases due to the lattice rotation relative to the origin point, which is considered to be the necessary precursor to PSN since the accumulation of misorientation by the rapid subgrain boundary migration should meet the requirement for the generation of high angle grain boundaries (HAGBs)^20^. Actually, the coarse unDRXed grains are observed to be subdivided into lots of subgrains surrounded by the low-angle grain boundaries as highlighted by hexahedrons in Fig. 4b. Figure 5b reveals dense dislocations accumulated at the boundary marked by the a red arrow, which indicates that more mobile dislocations should be generated during the subsequent further extrusion deformation and are trapped by the subgrain boundaries, then the subgrains transform to the DRXed grains. Therefore, it can be deduced that plate-shaped LPSO phases embedded in the α-Mg matrix promote the DRX process by PSN mechanism.

Figure 4e reveals that a small amount of fine DRXed grains form at the initial coarse grain boundaries of the Q + E sample, especially at the grain boundary surrounded by obvious color variation, namely orientation gradient (marked by a white circle). Such orientation gradient around the grain boundaries provides nucleation sites of DRXed grains^20^. At the mean time, bulging of the initial grain boundary can be observed, as marked by the black arrows, and the sizes of the bulges are comparable to the DRXed grain sizes. Therefore, discontinuous DRX also takes place at the initiation extrusion deformation of the Q + E sample. Further deformation brings about progressive DRX only in the mantle regions of the original coarse grains of the partially extruded Q + E sample, as shown in Fig. 4, so that the DRX ratio is much lower than the F + E sample (Fig. 4b). The dense nanoscale SFs precipitated on the basal planes of the coarse deformed grains (Fig. 4c) can effectively restrict the dislocation slips, especially the non-basal dislocations and grain boundary migration. As a result, although obvious color variations caused by the dense dislocations and/or subgrains can be observed inside the coarse grains in Fig. 4f, the strong pinning of the dislocations by the dense SFs impedes the transformation of subgrains into DRXed grains with HAGBs^21^,^22^.

**Figure 6:**
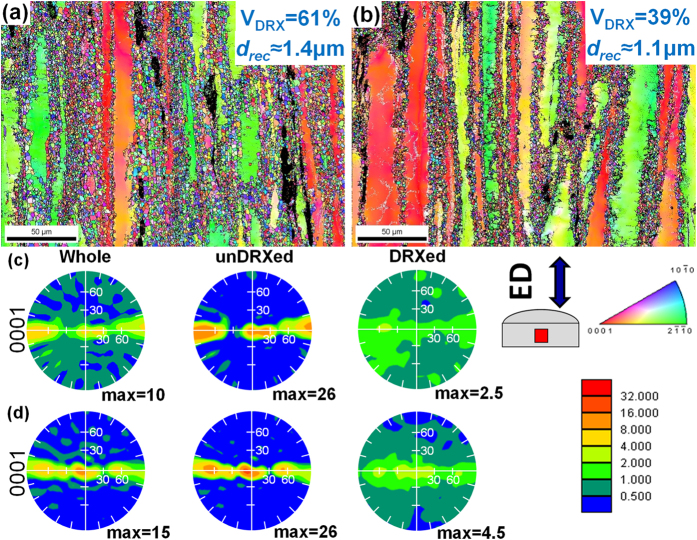
IPF maps and (0001) pole figures taken from the different regions of the as-extruded alloys (**a**,**b**) IPF maps, (**c**,**d**) pole figures, (**a**,**c**) F + E sample and (**b**,**d**) Q + E sample.

Figure 6 shows the IPF maps and (0001) pole figures of the as-extruded alloys. Because the plate-shaped LPSO phases in the F + E sample promote the DRX, while the dense SFs in the Q + E sample restrict the DRX, the F + E sample shows higher volume fraction of the DRXed grains than the Q + E sample, with the values of about 61% and 39%, respectively. As mentioned above that, the interspacing of plate-shaped LPSO phases plays a key role in the DRXed grain size, and hence the DRXed grains of the F + E sample are slightly larger than those of the Q + E sample.

### Texture evolution of the as-extruded alloys

Both the as-extruded alloys exhibit typical basal texture with basal planes parallel to the ED, as shown in Fig. 6c and d. The unDRXed grains have a stronger basal texture than the DRXed grains. The Q + E sample with higher volume fraction of unDRXed grains shows stronger texture than F + E sample. In addition, the unDRXed grains of both as-extruded alloys show identical texture intensities, whereas the DRXed grains of the F + E sample show weaker intensity than those of the Q + E sample. The (0001) pole figures inserted in Fig. 4a and c for the F + E sample and Fig. 4e for the Q + E sample are weighted by grain size and which demonstrate that the DRXed grains formed by the PSN mechanism show more random orientations than by the discontinuous DRX by grain boundary bulging. Robson *et al*.^20^ analyzed the effect of large particles on the recrystallization behavior of Mg-Mn alloys and they found that the texture of the DRXed grains formed by PSN is near random. However, that random texture minimally influences the final texture after complete recrystallization due to the small number of the PSN grains of their Mg-Mn alloys. On the contrary, high number density of the plate-shaped LPSO phases in the F + E sample provides sufficient sites for DRX by PSN and accordingly, texture of the DRXed grains are weakened than those in the Q + E sample. Therefore, it is an effective way to modify the DRX ratio and texture intensity of the Mg-Gd-Y-Zn-Zr alloys by controlling precipitation of LPSO phases and SFs.

### Mechanical properties of the as-extruded alloys

**Figure 7:**
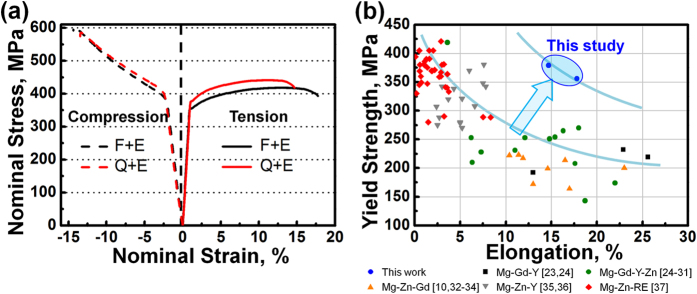
(**a**) Tension and compression nominal stress - strain curves of the as-extruded alloys, (**b**) tensile yield strength and elongation to failure of the extruded Mg-RE based alloys.

**Figure 8:**
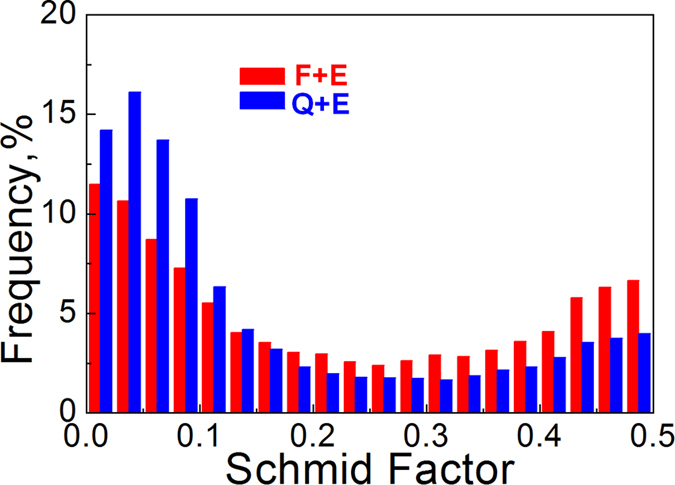
Schmid factor distribution histograms of the as-extruded alloys.

Figure 7a shows the tension and compression nominal stress-strain curves of the as-extruded alloys and the corresponding tensile/compressive yield strength (TYS,CYS), ultimate tensile/compressive strength (UTS,UCS) as well as the elongations to failure are summarized in Table 1. The F + E sample exhibits TYS of 356 MPa, UTS of 419 MPa and elongation to failure of 17.8%, while Q + E sample exhibits tensile yield strength TYS of 379 MPa, UTS of 442 MPa and elongation to failure of 14.7%. Figure 7b summarizes the tensile yield strength and elongation to failure of the existing extruded Mg-Gd/Y based alloys^11^,^23^,^24^,^25^,^26^,^27^,^28^,^29^,^30^,^31^,^32^,^33^,^34^,^35^,^36^,^37^, which suggests that both F + E and Q + E developed in this study show superior strength-ductility balance than other Mg-Gd/Y based alloys reported previously. The yield strength of Mg alloys at RT is mainly dependent on the activation of basal dislocation slip so that the high strength obtained in this study should be related to the facts as follows. At first, the fine DRXed grains with sizes of ~1 μm contribute to the high yield strength based on the Hall-Petch relationship. The nano-scale dynamically precipitated β phases exert effective Zener pinning on the grain boundaries and bring about dispersion strengthening. Since the Gd/Y atoms have high solid solubility in α-Mg matrix so that solution strengthening should also accounts for the high strength. Figure 8 shows the (0001) < 11
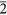
0> Schimid factor distributions of the as-extruded alloys, both of which have the trend to distribute towards the lower values, especially for the Q + E sample with higher volume fraction of unDRXed grains with strong basal texture. As a result, the basal slip should be obviously suppressed during the tensile test, leading to the strengthening when loading along the ED.

The increment in the CRSS for basal slip by precipitation is reported by Nie *et al*.^28^. The precipitation strengthening induced by basal plate-shaped LPSO phases (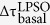) as well as SFs (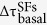) with large aspect ratios can be calculated by the same Eqs. (1)^28^:


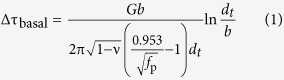


Where *G* is the shear modulus of the α-Mg matrix (about 16.6 GPa^29^), *b* is the Burger vector (0.32 nm for Mg^29^), ν is the Poisson’s ratio (*v *= 0.32), *f*_*p*_ is the volume fraction of the precipitates and *d*_*t*_ is the uniform diameter of the precipitates, which is estimated to be about 4.1 μm for the plate-shaped LPSO phases and 370 nm for the SFs. Because the plate-shaped LPSO phases spread across the whole unDRXed grains (Fig. 2a), the diameter of the phase should be higher than the thickness of the TEM sample of the F + E sample, and thus the volume fractions of the LPSO phase (*f*_LPSO_) is simply calculated by the area fraction based on TEM BF image in Fig. 3a. The volume fraction of the SFs (*f*_SFs_) with plate morphologies can be calculated by Eqs.(2)


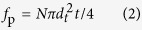


where *N* is the number density of the precipitates, *t* is the thickness of the precipitates, which is about 53 nm for the plate-shaped LPSO phases and 5 nm for the SFs. The number density of the plate-shaped LPSO phases in unDRXed grains of the F + E sample and SFs in the unDRXed grains of Q + E sample are estimated to be about 2.7 × 10^18^ m^−3^ and 3.2 × 10^20^ m^−3^, respectively using the convergent beam electron diffraction (CBED) technique^30^. Therefore, the 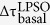 and 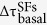 are calculated to be 2 MPa and 15 MPa, respectively. It can be seen that dense nanoscale SFs in the Q + E sample pin the basal dislocations more effectively than the plate-shaped LPSO phases in the F + E sample. However, the increments in the CRSS for basal dislocation slip by both of them are very low due to the fact that plates on the basal planes of α-Mg matrix provide poor barriers to the basal gliding dislocations. Recently, Kim *et al*.^31^ analyzed the effect of Y addition on the slip behavior in Mg using a molecular dynamics simulation and they found that Y addition increases the CRSS for all slip systems but reduce the difference in the CRSS between basal and <c + a> slip systems, which is supposed as one reason for experimentally reported activation of <c + a> slip in Mg-Y alloys^32^. In the present study, basal slip is remarkably inhibited in the unDRXed grains due to their low (0001) <11
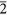
0> Schimid factor and which facilitates the activation of <c + a> dislocations during tensile test along ED. Besides, it is noted that extensive dislocations including non-basal < c + a> dislocations are introduced during extrusion process, as mentioned before. On the other hand, both the strong LPSO phases and SFs can effectively impede the non-basal slip dislocations that intersecting the basal planes, such as <c + a> dislocations then improve the strength of the alloys. For the as-rolled Mg-Gd-Y-Ag-Zr alloy sheet containing dense SFs, it is reported that the TYS increases with the decreasing interspacing of the SFs^33^. As a result, dense SFs should contribute much more than the plate-shaped LPSO phases to the yield strength of the alloys, due to the more effective pinning. At the mean time, the dislocations in the unDRX pile up and dislocation wall as well as sub-grain boundaries form during extrusion (Fig. 5a), which also serve as effective barriers to the dislocations during tensile test. On the other hand, the DRXed grains with relatively random orientations facilitate the basal slip when loading along ED, and hence the extruded alloys deform resembling the laminated metal composites with hard unDRXed layers and soft DRXed layers^11^,^34^ (Figs 2 and 6). Therefore, the unDRXed grains play a key role in the strengthening of the alloys and the tensile properties also demonstrate that Q + E sample with higher volume fraction of unDRXed regions exhibits higher strength than the F + E sample, which may suggest that the plate-shaped LPSO phases and SFs mainly influence the mechanical properties by controlling the DRX ratio.

However, the TYS of the F + E sample is only 23 MPa lower than that of the Q + E sample, despite that the volume fraction of DRXed grains for F + E sample is 22% higher than that for the Q + E sample. A high volume fraction of plate-shaped LPSO phases with high aspect ratio are also embedded in the DRXed regions and (Figs 3a,c and 6c). Their flat interfaces are parallel to the ED and they show strong anisotropy then behave as skeleton of the DRXed regions and reinforce the F + E sample^5^. The DRXed grains distributed between them are rarely pinned by β phases and act as ductile layers, so that these regions deform as multilayered composite. During tensile test loading along ED, these plate-shaped LPSO phases constrain the deformation of soft DRXed grains and carry higher stress concentration^35^, thereby improving the strength of the F + E sample more effectively. Consequently, the LPSO reinforced DRXed regions compensate the strength difference between the F + E and Q + E samples.

As mentioned above that the activation of non-basal dislocations is facilitated in the unDRXed grains of both the F + E and Q + E samples in the present study, which is beneficial for the ductility improvement of the as-extruded alloys. The tensile ductility is considered to be proportional to the interspacing between the precipitates: the smaller the interspacing, the lower the tensile ductility^36^. The dense SFs in the Q + E sample have much smaller interspacing than the plate-shaped LPSO phases in the F + E sample (Fig. 4), which make the unDRXed grains of the Q + E sample more fragile. The unDRXed grains mainly contribute to the strength but deteriorate the ductility, especially for the Q + E sample. On the other hand, the fine DRXed grains should play much more important role in the ductility of the as-extruded alloys with bimodal microstructure^11^. The strain localization is prone to present on the unDRXed grains due to the LPSO phases/SFs inside and the strong basal texture, but the strain localization can transfer to the ductile DRXed regions. In this way, the strain localization of the hard unDRXed grains can be effectively released and which promotes the homogeneous plastic deformation of the as-extruded alloys during tensile test^34^. Accordingly, superior strength-ductility balance is obtained for both alloys. Lower volume fraction of the ductile DRXed grains and smaller interspacing of dense SF in the Q + E sample leads to its lower elongation to failure.

Yield asymmetry with yield strength in tension higher than in compression was usually observed in as-extruded Mg alloys, which is caused by tension twining during compression along extrusion direction^37^. Yield anisotropies are significantly improved in both the F + E and Q + E samples with TYS/CYS of 1.07 and 1.06, respectively. The F + E and Q + E samples have almost same value of TYS/CYS although they have different DRX ratios (65% and 43%, respectively). This indicates that both LPSO and SFs in unDRXed regions restrict twinning during compressive deformation along ED^38^, and dislocation slip dominates in both tensile and compressive deformation of the two alloys.

In summary, the formation of plate-shaped LPSO phases and solute-segregated SFs was controlled by homogenization treatment, and the effect of LPSO and SFs on dynamically recrystallization (DRX) behavior of Mg-Gd-Y-Zn-Zr alloys was analyzed, and high performance Mg-8.2Gd-3.8Y-1.0Zn-0.4Zr alloys with superior strength-ductility balance have been successfully developed. Bimodal microstructure consisting of coarse unDRXed grains with strong basal texture and fine DRXed grains with relatively random orientations are observed in both alloys. The plate-shaped LPSO phases stimulate the DRX by particle stimulated nucleation mechanism so that high DRX ratio and weak texture is obtained in LPSO containing alloy. While for the alloy with dense fine SFs in the original grains, DRX is obviously restricted. Consequently the Mg-Gd-Y-Zn-Zr alloy containing dense SFs exhibits higher strength but lower ductility than the sample containing plated-shaped LPSO phases.

## Methods

### Material preparation

Mg-8.2Gd-3.8Y-1.0Zn-0.4Zr (wt.%) alloy ingot with 280 mm in diameter and 2940 mm in length was produced by direct chill casting. The specimens machined from the ingot were homogenized at 510 °C for 12 h in a Pyrex tube under an Ar atmosphere, then cooled in furnace to ambient temperature with speed of ~0.7 °C/min and immediately quenched in warm water of about 80 °C, respectively. The homogenized samples were machined to cylindrical samples with 43 mm in diameter and 50 mm in height for extrusion, then preheated at 400 °C for 5 min prior to the extrusion for homogenizing the temperature of the samples. The extrusion rods with 13.6 mm in diameter and 400 mm in length was produced by indirect extrusion at 400 °C with an extrusion ratio of 10:1 and a ram speed of 0.1 mm/s. The extrusion rods from furnace-cooled alloy and quenched alloy are denoted as F + E and Q + E, respectively.

### Microstructure characterization

The microstructure of the alloys was observed by Olympus BX60 M optical microscope (OM), JEOL JSM-7000F field-emission scanning electron microscope (FE-SEM) and JEOL JEM-2100F Transmission electron microscope (TEM) and high angle annular dark field scanning transmission electron microscopy (HAADF-STEM) operating at 200 kV. The texture was measured by an EDAX-TSL EBSD system operating at 25 kV, and the data were analyzed by OIM Analysis software.

### Mechanical property tests

The tensile specimens having a gauge length of 30 mm and a diameter of 6 mm and compressive specimens with a length of 15 mm and a diameter of 6 mm were machined from the as-extruded rods. The tensile and compressive tests with tensile and compressive directions parallel to the extrusion direction (ED) were conducted on a Shimadzu Autograph AG-I (50 kN) machine at an initial strain rate of 1 × 10^−3^ s^−1^ at room temperature (RT)^39^,^40^,^41^,^42^,^43^,^44^,^45^,^46^,^47^.

## Additional Information

**How to cite this article**: Xu, C. *et al*. Effect of LPSO and SFs on microstructure evolution and mechanical properties of Mg-Gd-Y-Zn-Zr alloy. *Sci. Rep.* **7**, 40846; doi: 10.1038/srep40846 (2017).

**Publisher's note:** Springer Nature remains neutral with regard to jurisdictional claims in published maps and institutional affiliations.

## Figures and Tables

**Table 1:** Tensile and compressive properties of the as-extruded alloys tested at RT.

	TYS, MPa	UTS, MPa	ε, %	CYS, MPa	UCS, MPa	ε, %	CYS/TYS
F + E	356 ± 2	419 ± 2	17.8 ± 1.0	380 ± 11	602 ± 8	14.3 ± 0.6	1.07 ± 0.3
Q + E	379 ± 1	442 ± 1	14.7 ± 0.8	401 ± 6	586 ± 3	13.4 ± 0.4	1.06 ± 0.2
